# Analysis of Alpha-2 Macroglobulin from the Long-Lived and Cancer-Resistant Naked Mole-Rat and Human Plasma

**DOI:** 10.1371/journal.pone.0130470

**Published:** 2015-06-23

**Authors:** René Thieme, Susanne Kurz, Marlen Kolb, Tewodros Debebe, Susanne Holtze, Michaela Morhart, Klaus Huse, Karol Szafranski, Matthias Platzer, Thomas B. Hildebrandt, Gerd Birkenmeier

**Affiliations:** 1 Institute of Biochemistry, University of Leipzig, Leipzig, Germany; 2 Department of Reproduction Management, Leibniz Institute for Zoo and Wildlife Research, Berlin, Germany; 3 Fritz Lipmann Institute–Leibniz Institute for Age Research, Jena, Germany; University of Saarland Medical School, GERMANY

## Abstract

**Background:**

The naked mole-rat (NMR) is a long-lived and cancer resistant species. Identification of potential anti-cancer and age related mechanisms is of great interest and makes this species eminent to investigate anti-cancer strategies and understand aging mechanisms. Since it is known that the NMR expresses higher liver mRNA-levels of alpha 2-macroglobulin than mice, nothing is known about its structure, functionality or expression level in the NMR compared to the human A2M.

**Results:**

Here we show a comprehensive analysis of NMR- and human plasma-A2M, showing a different prediction in glycosylation of NMR-A2M, which results in a higher molecular weight compared to human A2M. Additionally, we found a higher concentration of A2M (8.3±0.44 mg/mL *vs*. and 4.4±0.20 mg/mL) and a lower total plasma protein content (38.7±1.79 mg/mL *vs*. 61.7±3.20 mg/mL) in NMR compared to human. NMR-A2M can be transformed by methylamine and trypsin resulting in a conformational change similar to human A2M. NMR-A2M is detectable by a polyclonal antibody against human A2M. Determination of tryptic and anti-tryptic activity of NMR and human plasma revealed a higher anti-tryptic activity of the NMR plasma. On the other hand, less proteolytic activity was found in NMR plasma compared to human plasma.

**Conclusion:**

We found transformed NMR-A2M binding to its specific receptor LRP1. We could demonstrate lower protein expression of LRP1 in the NMR liver tissue compared to human but higher expression of A2M. This was accompanied by a higher EpCAM protein expression as central adhesion molecule in cancer progression. NMR-plasma was capable to increase the adhesion in human fibroblast *in vitro* most probably by increasing CD29 protein expression. This is the first report, demonstrating similarities as well as distinct differences between A2M in NMR and human plasma. This might be directly linked to the intriguing phenotype of the NMR and suggests that A2M might probably play an important role in anti-cancer and the anti-aging mechanisms in the NMR.

## Introduction

The naked mole-rat (*Heterocephalus glaber*) (NMR) living in East Africa is an eusocial colony building mammal (O’Rianin et al. 2008 Ecology of Social Evolution). Thereby, eusociality is mostly seen with insects like ants, bees, wasps, and others, the NMR is one of the rare known eusocial mammals–notably described so far only in the family *Bathyergidae*. NMR has a few unusual features compared to other mammals. The NMR is a very long-lived rodent species, which has a lifespan of over 30 years [1]. This suggests specific aging mechanisms, which are accompanied or potentially caused by cancer resistance. So far, no tumor was ever observed in the NMR [2]. There is strong evidence that the longevity of NMR is mainly maintained by the cancer resistance, because neoplasia is the primary cause of death in other mammalian species like mice [3]. There is an emerging interest to bring in line the longevity and cancer resistance by identifying underlying molecular mechanisms to understand the most fascinating and extraordinary NMR phenotypes.

Previously, a handful articles had been published, giving hints and trials to explain those mechanisms in the NMR [4–8]. Thereby social and biological/biochemical features are adducted. From a social point of view the eusocial mode of life with a cooperative care of the offspring and the intergenerational propagation of skills [2] as well as living in a group is widely associated with a longer life [9]. Another health supporting effect is associated with the underground life. Those animals are protected from extreme climate conditions and predators, which favors longevity and a lower mortality rate [2, 10]. On the cellular and biochemical level NMR exhibit several unique anti-tumor features like slow cellular growth, effective contact inhibition, formation of high-molecular-mass hyaluronan and optimized protein synthesis [11].

Alpha-2 macroglobulin (A2M) is a major extracellular protein in the blood. Recently, A2M transcript levels were shown to be increased in the NMR liver compared with that of mice by 140-fold [12]. So far, NMR-A2M protein is not further characterized. Its human counterpart is a homotetrameric protein of 720 kDa playing a role in maintaining homeostasis of cytokines and growth factors [13]. The function of A2M in humans is partly different compared to rodents (e.g. mice, rats and rabbits), where A2M is a major acute phase protein [14]. In general, A2Ms from different species are very well described and briefly characterized in a review by Sottrup-Jensen [15]. Human A2M is able to bind a very wide range of cytokines, growth factors, especially TGF-ß1, TNF-alpha and IL-1ß and hormones [16–18]. Another important function is the capability to inactivate a great variety of proteinases, like trypsin, chymotrypsin, elastase or metalloproteinases. Upon binding of proteinases, A2M undergoes a major conformational change, which results in expression of previously hidden receptor binding sites on its surface. This enables the so-called “transformed A2M” (A2M*) to bind to its specific receptor, named LRP1 (CD91) [19, 20]. Ligation of LRP1 induces the receptor-mediated rapid clearance of the A2M-proteinase-complexes from the blood and tissue [21]. Other proteins like growth factors and cytokines are bound reversibly to A2M. Thereby, A2M fulfills important functions with respect of the tissue homeostasis of those molecules [22, 23].

A2M is suggested to play an important role in cancer and aging [24, 25]. The human A2M blood concentration is negatively correlated with age, decreasing from approximately 4 mg/mL at birth to 1.5 mg/mL in the elderly [26]. Therefore, its function in blood homeostasis and age related diseases are of great clinical and geriatric interest. Key factors responsible for malignancy involve also adhesions molecules. Since it is known that the NMR is cancer resistant, those are of detrimental interest and a deeper analysis of adhesion molecule expression and function in the NMR is warranted. For example, the transcript level of the epithelial adhesion molecule EpCAM was found to be increased in the NMR liver by 290fold compared to mice, which provides strong evidence, that cell coherence and integrity is influenced by the expression of adhesion molecules [12].

In the present study, we analyzed plasma A2M from NMR in comparison to its human homologue. For the first time we could show an elevated plasma protein level of NMR-A2M and similarities as well as distinct differences of the molecular structure and function compared to human A2M. Furthermore, we surprisingly found NMR plasma to increase cell adhesion in human fibroblasts. In addition, we described and annotated the NMR-A2M accordingly to the human-A2M protein with post-translational modifications and could identify similarities and differences, which could play a role in NMR-related peculiarities.

## Results

### In silico sequence analyses

Searching for NMR-A2M sequences in the relevant databases resulted in two available mRNA sequences. The first (Ref.Seq.: NM_001279851.1; Uniprot: E3VX34_HETGA) was described by Szafranski et al. (database entry) and a second was predicted by genomic sequencing of the NMR genome (GenBank: JH171302.1; UniProt: G5BPM1_HETGA) [27]. Only the transcript of NMR-A2M had been verified and described so far. The existence of the NMR-A2M protein was only predicted. The sequence of NMR-A2M yielded 1475 (Uniprot: E3VX34_HETGA) and 1595 [27] amino acids, respectively, resulting in a calculated molecular mass of 162.519 kDa and 175.364 kDa, respectively. Comparing these findings with the human A2M sequence, the NMR sequence described by Szafranski et al. (2010) is more similar than that of Kim et al. (2011). Therefore, all following analyses were done with the more similar NMR-A2M sequence compared to the human one (UniProt: E3VX34) (Table 1).

**Table 1:** Comparative analysis of the human and naked mole-rat (NMR) alpha 2-macroglobuolin sequence.

	human	NMR (1)	NMR (2)
**Amino acids**	1474	1475	1595
**Mol. weight [kDa]**	163.291	162.519	175.364
**Identity RNA**		85%	
**Identity protein**		98%	
**UniProt ID**	P01023	E3VX34	G5BPM1
**Protein existence**	evidence at protein level	evidence at transcriptional level	predicted
**References**	several	Szafranski et al. 2010 database entry	Kim et al. 2011

**Fig 1:**
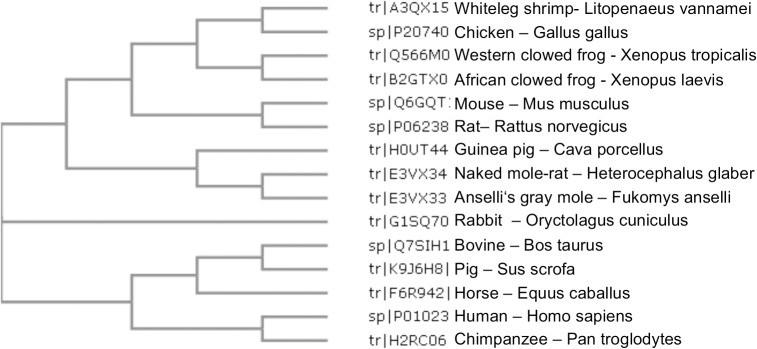
Phylogenetic tree of A2M. Phylogenetic analysis of A2M protein sequences was done using the ClustalW2 onlinetool (https://www.ebi.ac.uk/Tool/phylogeny/clustalw2phylogeny). The compared A2M protein sequences were readout from the Uniprot database.

NMR-A2M has an overall mRNA identity of 85% and a similarity for the protein sequence of 98% to human A2M. Phylogenetic analysis by ClustalW2 resulted in a close relationship of the NMR-A2M to Ansell’s mole rat and Guinea pig A2M (Fig 1).

The human A2M as well as the NMR-A2M have a signaling peptide sequence (aa position 1–23) at the N-terminus, which was annotated by similarity. The bait region, which is a hallmark of A2M in all species, is located in the NMR at position 688 to 738. It contains three trypsin cleavage sites at the arginine’s 703, 711 and 729 (Table 2). The analysis of the N-glycosylation sites resulted in 10 potential N-glycosylated amino acids in the position 55, 70, 263, 396, 410, 870, 992, 1078, 1367, and 1427. Thereby, the NMR-A2M shares 7 N-glycosylation sites with the human A2M, which has eight predicted N-glycosylation sites. The N-glycosylation site at position 247 of the human A2M is not present in the NMR-A2M. All disulfide bridges in the human A2M are identified in the NMR-A2M by similarity (Table 2). The potential iso-glutamyl lysine isopeptide cross-link at position 693 in the human A2M could be found in the NMR-A2M at the respective position 694 (Table 2). The Cys972 and Gln975 responsible for the thiolester binding in the human A2M are located at position Cys973 and Gln976 in the NMR-A2M.

**Table 2:** Protein sequence features of A2M from *Heterocephalus glaber*.

*Feature key*	*Position(s)*	*Length*	*Description*
**Molecular processing**			
Signal peptide	1–23	23	by similarity
Chain	24–1475	1452	Szafransky et al.; by similarity
**Regions**			
Region	688–738	51	Bait region (potential)
Region	703		Trypsin cleavage side
Region	711		Trypsin cleavage side
Region	729		Trypsin cleavage side
**Amino acid modifications**			
Glycosylation	55		by similarity
Glycosylation	70		by similarity
Glycosylation	263		potential
Glycosylation	396		by similarity
Glycosylation	410		by similarity
Glycosylation	870		by similarity
Glycosylation	992		by similarity
Glycosylation	1078		potential
Glycosylation	1367		potential
Glycosylation	1427		by similarity
Disulfide bound	48←→86		by similarity
Disulfide bound	251←→299		by similarity
Disulfide bound	269←→287		by similarity
Disulfide bound	278		interchain with 431 (by similarity)
Disulfide bound	431		interchain with 278 (by similarity)
Disulfide bound	470←→560		by similarity
Disulfide bound	592←→772		by similarity
Disulfide bound	641←→688		by similarity
Disulfide bound	822←→850		by similarity
Disulfide bound	848←→884		by similarity
Disulfide bound	922←→1322		by similarity
Disulfide bound	1080←→1128		by similarity
Disulfide bound	1353←→1468		by similarity
Cross-link	694		Isoglutamyl lysine isopeptide (Gln-Lys) (interchain with K-? in other proteins); potential by similarity
Cross-link	973←→976		Isoglutamyl cysteine thioester (Cys-Gln); by similarity and this publication

### Plasma composition

Different variations of polyacrylamide gel electrophorese (PAGE) were done to investigate the protein distribution of the NMR plasma in general and the presence of characteristic features of the NMR-A2M in particular.

**Fig 2:**
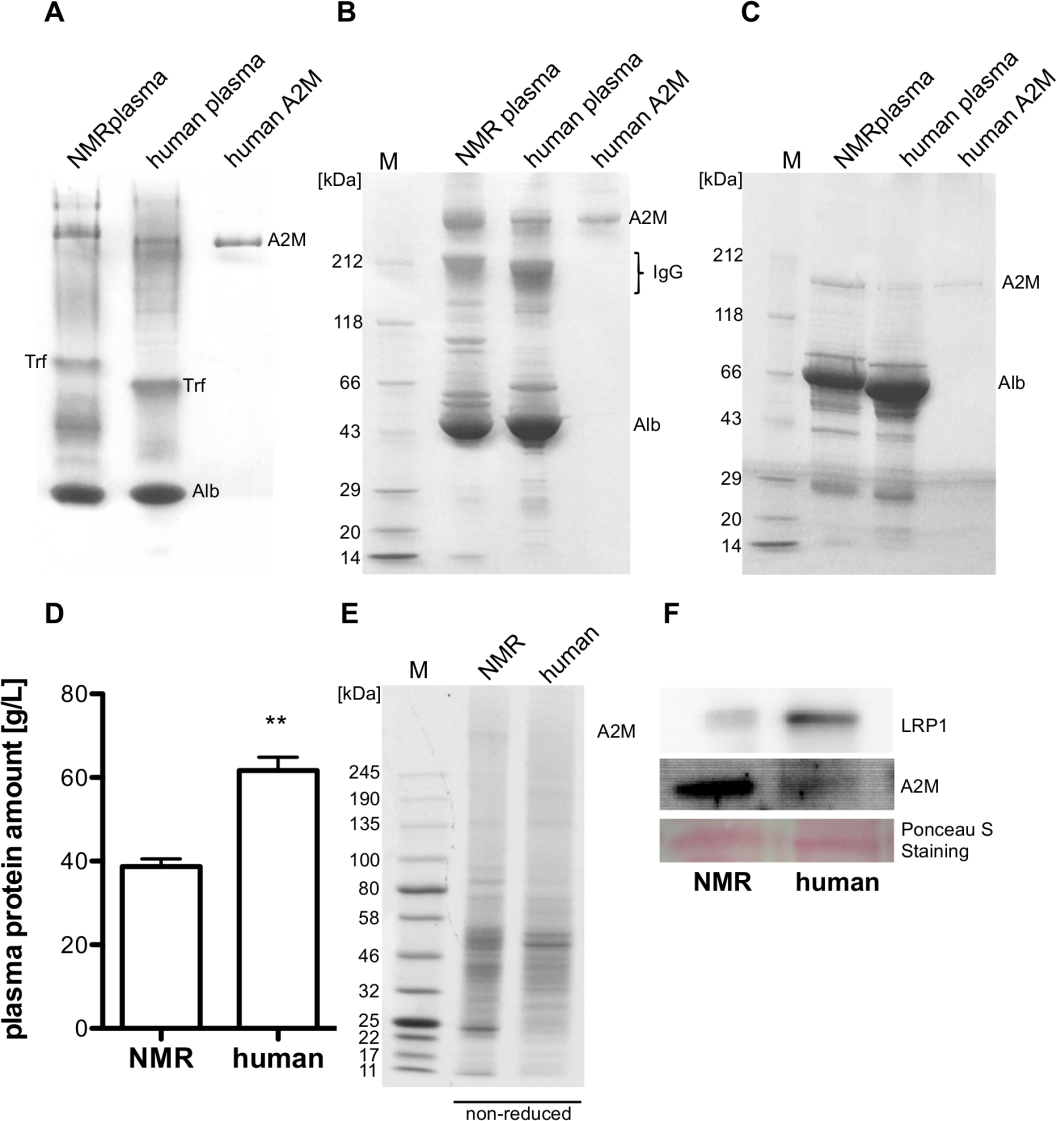
Plasma and liver protein analysis. NMR, human plasma (30μg) and purified human A2M (2.5μg) were separated by native PAGE (4–20%) (A) and SDS-PAGE (4–20%) under non-reducing (B) and reducing conditions (C). The protein concentration of NMR and human plasma was determined according to Bradford (**—p<0.01) (D). Protein extracts from human and NMR liver (20 μg each) were separated by SDS-PAGE (4–20%) (E) and subjected to western blot analysis using a polyclonal rabbit anti-human A2M antibody (5 μg/mL) and a monoclonal mouse ß-subunit specific anti-human LRP1 antibody (10μg/mL) (F). (A2M –alpha-2 macroglobulin, Alb–albumin, IgG–immunoglobulin, Trf—transferrin).

Native polyacrylamide gradient gel electrophoresis (native PAGE) showed NMR-A2M to run at a position appearing to have a lower electric mobility than A2M in human plasma and purified human A2M, which might indicate a higher molecular mass (Fig 2A). Native human A2M is a tetramer with a molecular mass of 720 kDa. Using SDS-gradient PAGE (4–20%) under non-reduced conditions NMR-A2M moves as a dimer with an approximate molecular mass similar to human A2M (360 kDa) (Fig 2B). Under reduced conditions NMR-A2M is cleaved to monomers of 180 kDa similar to human A2M (Fig 2C). Furthermore, the overall NMR plasma protein concentration was found to be lower than in human (38.7±1.79 mg/mL *vs*. 61.7±3.20 mg/mL; n = 5) (Fig 2D). Human plasma seems to contain a higher content of immunoglobulins (Fig 2A and 2B) and the overall protein pattern seems to be different, too. NMR in contrast to human plasma displays different protein bands (distance between Alb and IgG) in non-reduced SDS-PAGE (between marker 43 kDa and 212 kDa). At least in human plasma, haptoglobin- and Gc-group protein genetic variants move at this position. Nonetheless, an overall analysis of the three different types of electrophoresis displayed stronger band intensities of A2M in the NMR plasma compared to human plasma, indicating a higher A2M content in NMR.

The most abundant protein in the NMR plasma is albumin like in human plasma. Transferrin with a molecular mass of 80 kDa in humans was less migratory in NMR, indicating different molar masses or variations in glycosylation.

Protein and immunoblot analysis of liver extracts from NMR and humans revealed a high number of low molecular mass proteins for both species. A remarkable difference is the appearance of a high molecular mass protein species in NMR liver (Fig 2E). This protein band was shown to react with antibodies against human A2M by western blot (Fig 2F) showing a higher A2M protein amount in NMR liver extract than in human. In contrast, a lower amount of immunoreactive LRP1 was detected in the NMR liver extract compared to human (Fig 2E).

### A2M activation

**Fig 3:**
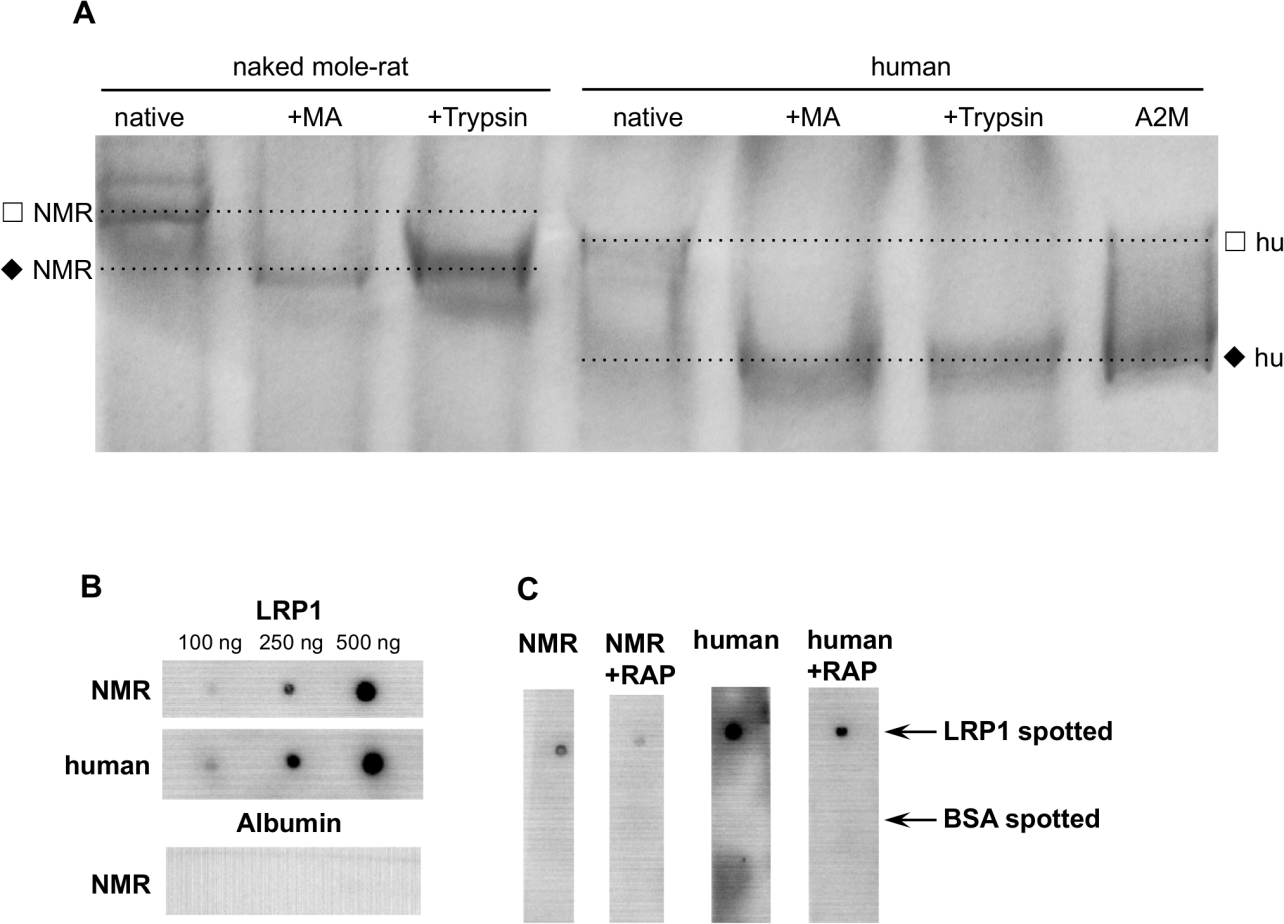
RATE electrophoresis of NMR and human plasma and analysis of the receptor-binding properties of their A2M. RATE electrophoresis was done using 50 μg native NMR and human plasma and 50 μg of methylamine and trypsin treated plasma, respectively. Isolated human A2M served as control. Methylamine as well as trypsin shift human and NMR-A2M from the slow- to the fast-moving form. To visualize the respective protein bands the gel was stained with Commassie (A). Purified LRP1 (100–500 ng) was spotted to a nitrocellulose membrane and incubated with either 10 μg/mL human or NMR plasma. BSA served as negative control. A2M binding to LRP1 was detected by a polyclonal rabbit anti-human A2M antibody (5 μg/mL) (B). To block the binding of A2M to LRP1 the spotted LRP1 was pre-incubated with 1.5 μM RAP for 30 min prior to the addition of human or NMR plasma (C). (☐ - native/slow form of A2M, ◆ –transformed/fast form of A2M).

A2M is known to occur in two different conformational states. Binding of proteinases, results in a conformational change toward a more compact structure of the protein. *In vitro*, such a conformational transition of A2M can be obtained also by the treatment of A2M with methylamine, which is known to cleave A2M’s thioester bond and thus triggering a major conformational change similar to proteinase treatment. The conformational change can be visualized by the so-called rate PAGE (Fig 3A). This method allows discrimination between the two different forms of A2M, the slow- (native form) and the fast-migrating (activated form with the cleaved thioester bond) form, which is said to be caused due to changes in globularity [28]. Binding of trypsin as well as reaction with methylamine showed similar moving pattern, depending on the completeness of the conformational change, in both, NMR and human plasma. Overall, A2M from NMR was shown to be reactive toward methylamine and trypsin thus showing similar functional properties as its human analogue. However, the conformational convertibility seems to be more complex in NMR-A2M indicated by the appearance of protein bands between the positions of the slow and fast forms of human A2M.

### Receptor binding of A2M from NMR and human plasma

Human A2M* (transformed A2M) is known to bind specifically to soluble or immobilized LRP1. For that purified human LRP1 was spotted to a nitrocellulose membrane and incubated with human or NMR plasma, respectively (Fig 3B). As seen, A2M* from both species bind to the immobilized receptor indicating the presence of receptor-binding domains in NMR-A2M. In contrast, no binding was observed to immobilised albumin corroborating the specificity of interaction. The receptor associate protein–RAP is known to block binding of diverse ligands to LRP1. Indeed, RAP was found to diminish the interaction of NMR-A2M* to LRP1 (Fig 3C). The results indicate that A2M from NMR contains conserved region at the C-terminal domain capable of binding to human LRP1.

### A2M quantification and immunological detection

**Fig 4:**
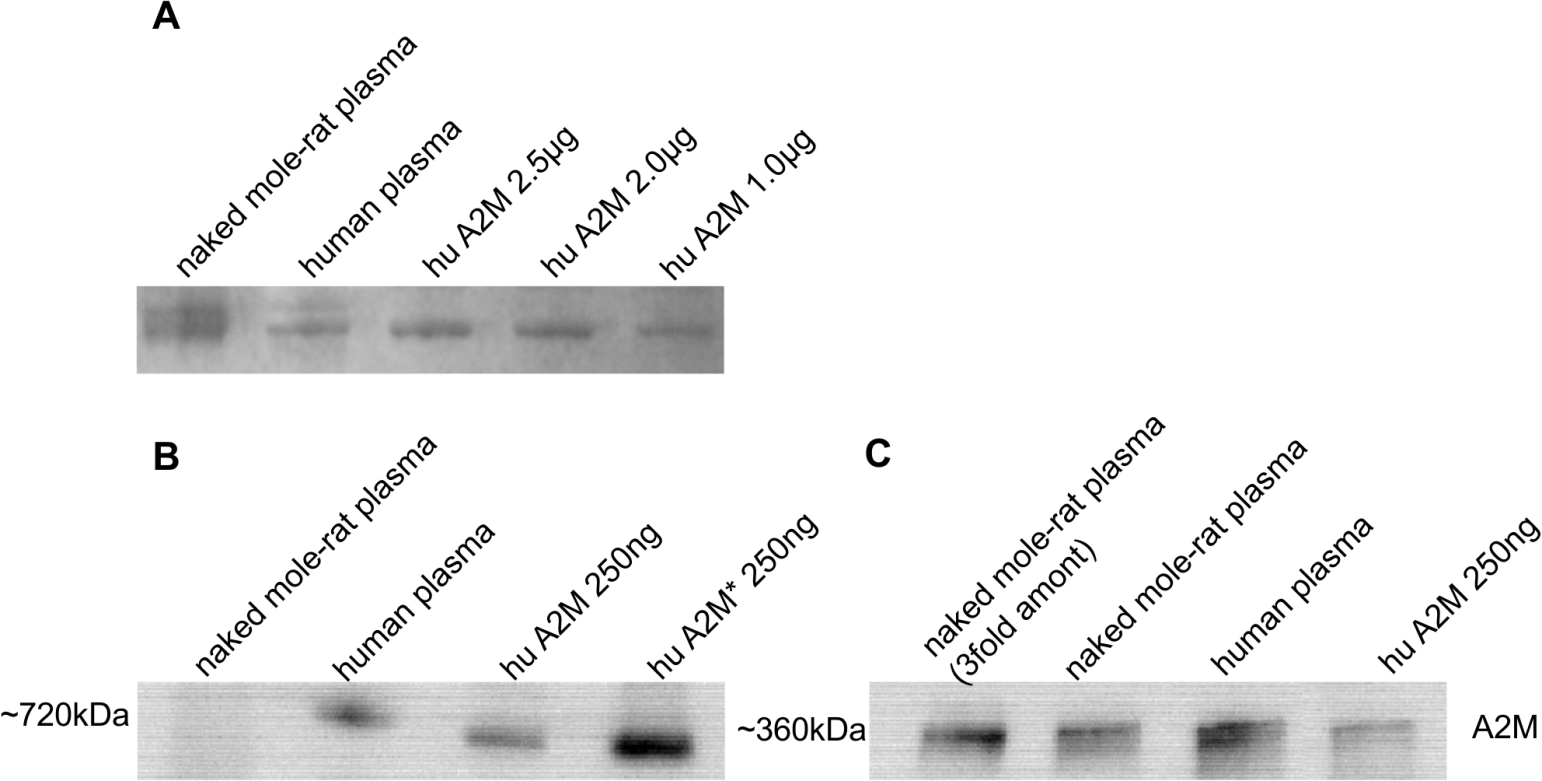
Western blot of plasma from NMR and human for alpha-2 macroglobulin. The determination of A2M in plasma samples was performed by electrophoresis in 7% SDS-gel using NMR and human plasma, 30 μg each, and purified human A2M (1–2.5 μg). A2M was quantified by Commassie staining using isolated human A2M to prepare a calibration curve (A). Western blot analyses using a native gradient PAGE (4–20%) was applied to detect the transformed form of A2M using the alpha-1 antibody (B) and a 7% non-reducing SDS-PAGE to detect A2M with a polyclonal antibody using 9 and 3 μg NMR or 3 μg human plasma and 250 ng isolated human A2M (C).

A 7% SDS-PAGE was used to evaluate the concentration of A2M in plasma of NMR and human. Purified human A2M served as internal standard. The Coommassie Blue stained gels were scanned and analyzed using ImageJ software. 30 μg NMR and human plasma protein, respectively, was loaded to the gels. Generating a standard curve with purified human A2M, the calculated A2M concentration in NMR and human plasma was 8.3±0.44 mg/mL and 4.4±0.20 mg/mL, respectively (n = 5). Correspondingly, A2M represents 6.9±0.37% of total plasma protein content in humans and 15.3±0.70% of total plasma protein content in the NMR (Fig 4A). In addition, the immunoreactivity of NMR-A2M was checked using a conformation specific monoclonal mouse anti-human A2M antibody (alpha-1), known to recognize a spatial C-terminal epitope exposed only in transformed A2M (Fig 4B) and a polyclonal rabbit anti-human A2M antibody (Fig 4C). For testing the monoclonal antibody, the samples were run obligatory in native gradient gels before blotting, because SDS is known to modify the spatial structure of A2M. As seen, the monoclonal antibody detected the transformed human A2M (A2M*), but no reactivity was observed toward NMR-A2M (Fig 4B). On the other hand, the polyclonal rabbit antibody expectedly detected human as well NMR-A2M (Fig 4C), the latter with obviously lower reactivity because approximately 3fold the amount of NMR-A2M from plasma is needed to get a comparable immunological signal intensity as with human A2M (Fig 4C). These results corroborate the data obtained by densitometric analysis of stained protein bands (Fig 4A).

### Anti-proteolytic and proteolytic activity of human and NMR plasma

**Fig 5:**
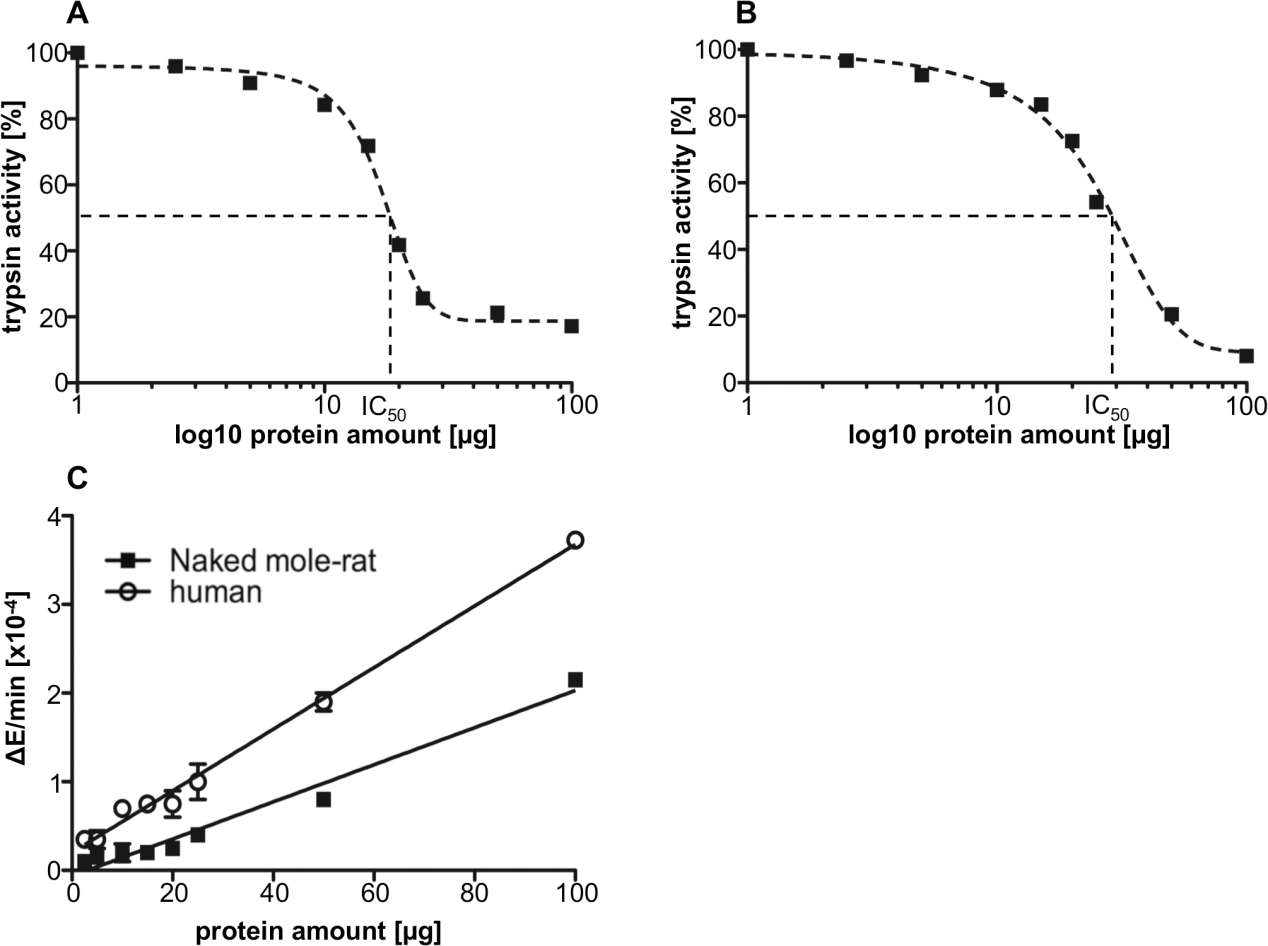
Anti-tryptic and proteolytic activity of NMR and human plasma. The inactivation of 0.05 μg trypsin was titrated with increasing amounts of human (A) or NMR (B) plasma corresponding to 1–100 μg protein and measured by the conversion of BAPNA to p-nitroaniline at 405 nm. The intrinsic tryptic activity of NMR and human plasma was determined by measuring the conversion of BAPNA to p-nitroaniline induced by 1–100 μg plasma protein (C). (*—p<0.05)

Plasma of all mammals and most likely NMR contains different proteinase inhibitors and proteolytic enzymes involved in specific pathways like blood coagulation/fibrinolysis and the complement system or serve general functions. It is known that A2M binds and inactivates proteinases of all classes and specificities. Therefore, it was of interest to analyze the anti-proteolytic activity of whole plasma by using trypsin as reference enzyme. The trypsin-inhibitory capacity was measured by calculating the amount of plasma capable to inhibit 0.05 μg trypsin. As shown in Fig 5, the inhibitory capacity at lower protein content (2.5–20 μg) was higher in human than in NMR plasma, represented by the IC_50_ of 17.08 μg for human and 25.11 μg for NMR plasma. However, at higher plasma protein concentrations a higher overall inhibitory activity was observed in the NMR plasma compared to human plasma. There was less residual tryptic activity in NMR plasma (8.06%) compared to human plasma (17.22%) by using 100 μg plasma protein to inhibit 0.05 μg trypsin (Fig 5A and 5B).

The intrinsic proteolytic activity of human and NMR plasma was determined by cleavage of BAPNA to p-nitroaniline. NMR plasma has a significant lower endogenous proteolytic activity than human plasma (Fig 5C). Reflecting 100 μg NMR plasma less proteolytically active (approximately 40%) than equivalent amount of human plasma. This might be explained by the higher concentration of the main proteinase inhibitor A2M.

### Adhesion molecules under NMR-A2M treatment

**Fig 6:**
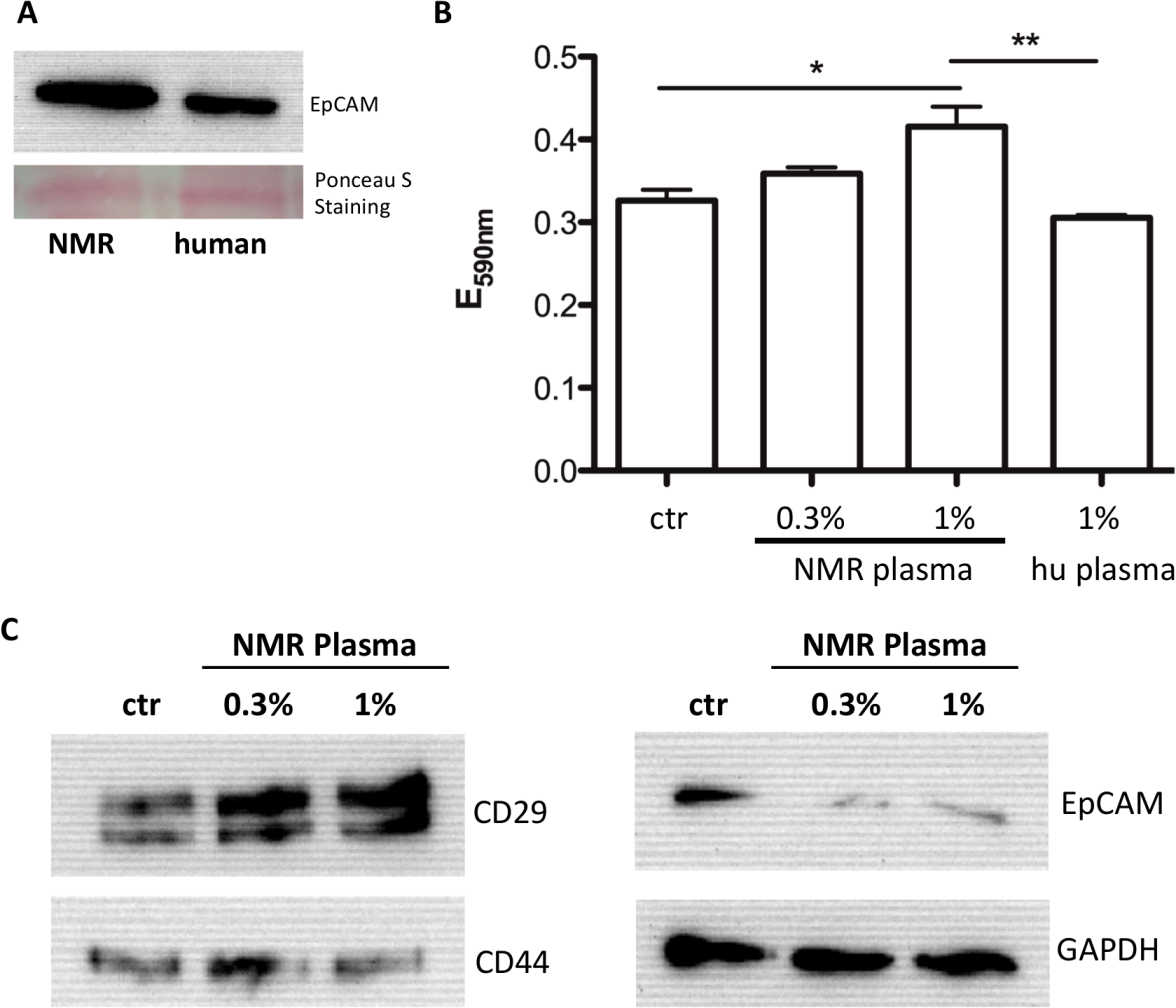
Adhesion induction by NMR plasma supplementation in human fibroblasts. The determination of the EpCAM protein in NMR and human liver samples were analysed by electrophoresis in 10% SDS-gel using 20 μg protein in conjunction with immunoblotting using a monoclonal mouse anti-human EpCAM antibody (A). Human fibroblasts were cultured in medium supplemented with 0.3 or 1% NMR plasma for 24h and, controls were supplemented with PBS (ctr) or 1% human (hu) plasma), respectively. Cells were treated by trypsin/EDTA solution for exactly 1 min, washed and the remaining adherent cells were stained by Gentiana solution. The absorbance of the released dye is directly proportional to the number of adherent cells (B) (**—p<0.01; *- 0.05). Western blot analysis for CD29, CD44, EpCAM and GAPDH was performed by electrophoresis in 8% SDS-gel using 10–25 μg protein after 0.3 or 1% NMR plasma supplementation for 24h. CD29, CD44, EpCAM and GAPDH were detected with respective monoclonal antibodies (C).

Next generation sequencing data already indicated high expression of adhesion molecules in NMR liver [12]. We could corroborate these results at protein level and found higher expression of EpCAM protein in NMR liver extract compared to human (Fig 6A). Next we hypothesized that components of the NMR plasma may modulate the expression of cell adhesion molecules and thereby cell adhesion. Therefore, we analyzed the adhesive property of cultured human fibroblasts in a so-called trypsination-adhesion assay. Culture medium of human fibroblasts was supplemented with 0.3 or 1% NMR plasma. Supplementation with PBS or 1% human plasma served as controls. A significant increase in cell adhesion was observed upon supplementation with 1% NMR plasma (Fig 6B). To target the question, which adhesion molecules could be responsible for the increased adhesion, we analyzed CD29, CD44 and EpCAM by western blot analysis in controls and in 0.3 and 1% NMR plasma treated human fibroblasts. We observed an increase in CD29 protein amount when the fibroblasts were treated with 0.3 and 1% NMR plasma. On the other hand, CD44 levels did not change under the same treatment. EpCAM was expressed on a very low amount in the fibroblasts but diminished further under NMR plasma treatment (Fig 6C).

## Discussion

In the present study we performed a comparative analysis of plasma components from the long-lived rodent NMR with that from humans. Because of several peculiarities of the NMR, like cancer resistance and longevity these investigations are of high scientific interest. From our results we suggest that some distinguishing features could at least in part explain the unusual properties of the NMR.

Recent gene expression analysis in the liver of NMR and mice demonstrating 660 genes significantly 5fold overexpressed [12]. Most prominent expression was related to the plasma proteinase inhibitor A2M and proteins involved in cell-cell interaction and ROS defense mechanism. To elucidate cancer resistant mechanisms we compared two long-lived species, the NMR and humans, to illustrate potential anti-cancer mechanisms for human well being.

In a former study we have shown that aging is accompanied with a significant decline of A2M level in blood [26]. At present, numerous studies describe the outstanding function of A2M in regulation of cell and tissue homeostasis. This protein is unique as it inhibits proteinases of all classes, is involved in metabolism of disease-related growth factors and cytokines and in the pathogenesis of various diseases such as cancer, Alzheimer`s disease, infection and inflammation [24, 29–31].

These findings prompted us to focus on analysis of structural and functional properties of A2M from both species based on the hypothesis that the NMR data can provide substantial contributions to identify anti-cancer strategies in humans.

Various functions of A2M are mediated through binding to its receptor, LRP1. This huge transmembrane protein (600 kDa) is involved in pathogenesis of atherosclerosis, cancer cell migration and invasion, lipid metabolism as well as clearance of the Alzheimer peptide amyloid ß [32–35]. LRP1 binds more than 30 different ligands and displays the fastest and most effective clearance system of protein ligands from blood and tissues. Recently, it was shown that leptin forms complexes with A2M and is cleared from the blood by receptor-mediated endocytosis through LRP1. Computer simulation of this ternary interaction revealed that the stationary concentration of plasma leptin is strongly affected by the level of A2M [36]. This is of importance because other growth factors like TGF-ß1 and VEGF causally involved in tumor progression also bind to A2M and are cleared by the same mechanism. Furthermore, LRP1 act as co-receptor for numerous signal receptors involving in the wnt/ß-catenin pathway, the uPA/uPAR system and others [37]. As an important inhibitor of tumor-associated metalloproteinases and regulator of the urokinase-type plasminogen activator (uPA) system in cancer, A2M controls tumor cell migration and invasion [38]. These few examples may display the importance of the A2M-LRP1 axis in regulation of tissue and blood homeostasis.

For the first time we identified A2M in NMR by direct comparison with human A2M and by immunological methods. We found that NMR plasma contains approximately two to three times higher level of A2M compared to human plasma. Testing cross-reactivity of NMR-A2M with a panel of anti-human A2M monoclonal antibodies we failed to see any reactivity. Even the receptor-binding-domain (RBD) specific antibody, alpha-1, known to react only with transformed A2M, showed no binding. We recently found that this antibody recognizes the consensus peptide sequences, S1349-R-S1351 … D1330-E-P-K1333, two separated epitopes comprising a conformational epitope within the RBD of human A2M [39]. The absence of binding to NMR-A2M was most probably due to amino acid exchanges within the split epitope to N1350-R-P1352 … D1331-G-P-K1334 in NMR, replacing 3 of 7 amino acids at positions 1, 3 and 5 of the epitope. In contrast, the binding of NMR-A2M to its specific receptor (LRP1) as experimentally proved could be expected, because the two essential lysine residues at position 1395 and 1402 (NMR: Lys1393 and Lys1400) and the loupe stabilizing Cys1355 and Cys1471 of human A2M (NMR: Cys1353 and Cys1368) are present in the NMR-A2M [40]. The predicted eight beta-sheets and one alpha-helix can be found by similarity in the NMR-A2M [20]. Most structures found in the human A2M were also present in the NMR (disulfide bridges, N-glycosylation, bait-region, trypsin-binding sites). However, prediction of N-glycosylation sites in the NMR-A2M revealed two additional sites (Table 2). While the human A2M has 8 N-glycosylation sites, the NMR protein has 10. This could be an explanation for the higher molecular weight of NMR-A2M seen in the native PAGE (Fig 1A), since this could not be explained solely by the NMR-A2M amino acid composition. However, also other modification, like O-glycosylation can contribute to this phenomenon, since O-glycosylations are the mostly occurring and most complex modifications in eukaryotes with a species-specific fashion.

One potential mechanism responsible for the extreme cancer resistance in NMR was previously shown [5]. A high-molecular-mass hyaluronan (HA) was identified, which is secreted by NMR fibroblasts but not by fibroblasts from humans or mice. As long as these cells produced HA they were prevented from malignancy. Knocking down of the HA synthesizing enzyme (HAS2) or overexpression of the degrading enzyme (HYAL2) resulted in increased malignancy of NMR fibroblasts. The texture, composition and stability of the extracellular matrix are determining hallmarks in malignancy. The major receptor for HA in human and mouse is CD44. Blocking CD44 caused cultured NMR cells to grow faster [5]. Recently, it was shown that LRP1 binds to CD44 and thus regulates the adhesive properties of tumor cells [41]. Our findings that NMR-A2M binds to LRP1 and that this binding was interfered by RAP may shed light on the possible role of A2M in this interplay. Culturing human fibroblasts with 1% NMR plasma showed an increase in adhesion of these cells in comparison to the addition of human plasma. A western blot analysis displayed CD29 to be increased under NMR plasma supplementation (Fig 6C). We could not observe changes in CD44 expression in human fibroblasts upon NMR-plasma treatment that would explain the observed increase in cell adhesion. One reason could be that other cell adhesion molecules such as the integrin CD29. The primary function of integrin family members is to mediate cell-cell and cell-matrix adhesion. NMR-plasma increased the expression of CD29, which could therefore stabilize these interactions rendering fibroblast less sensitive to trypsination. EpCAM (CD326) is expressed by the epithelium of healthy individuals but overexpressed in most carcinomas. In most tumors high EpCAM expression was associated with metastasis and poor prognosis. However, for different tumor types contradictory results have been reported [42]. Nevertheless, it has been reported that ß-catenin activation induced EpCAM transcription via binding of TCF/Lef transcription factor to the EpCAM promotor [43]. This is of interest as we could recently show that A2M inhibits wnt/ß-catenin pathway in tumor cells [24]. This could explain the inhibiting effect of A2M on EpCAM expression (Fig 6C).

Therefore, we deduced that a major factor responsible for the increased cell adhesion upon NMR plasma exposure of human fibroblasts could be A2M.

The higher amount of A2M mRNA found in NMR liver (140fold) compared to mice [12] and the higher protein content of A2M in NMR plasma (2-3fold) compared to human as found in this study is striking indeed. It has been recently shown that A2M is capable to mediate anti-cancerous effects by fostering tumor antigen presentation in tumor bearing mice [44]. Furthermore, A2M was found to mediate clearance of the TGF-ß1 due to its high affinity binding. For many tumor entities this factor is known to sustain tumor growth [45]. The finding that the level of A2M decreases with age [26] may probably account for defects in clearance of many tumor-promoting factors and aging-related peptides in human [46].

These drawbacks seem to be counteracted in NMR by higher level of expression of this protein. No data are available showing an age-dependent change in the level of NMR-A2M like in humans [26]. The abundance of A2M-mRNA in the NMR liver compared to its moderate increased plasma level is indicative to a high turnover of the protein in tissue and blood provided that A2M transcripts are fully translated. Thus, it is suspected that NMR-A2M at least in part accounts for the fascinating resistance of NMR against tumors.

Recently, it was convincingly demonstrated that administration of A2M incorporated in microparticles protected mice against hypothermia, inflammation and diminished the bacterial load in a sepsis animal model [29]. This is of interest as NMR is unable to sustain thermogenesis [47].

Furthermore, A2M is the only plasma proteinase inhibitor capable to inhibit proteinases of all four classes and regulate proteinase activity during inflammatory events. NMR is obviously exposed to many bacterial infections during his underground life in the soil. Thus, the high A2M level might be an early defense mechanism against bacterial proteinases. High concentrations of this protein were also found in horseshoe crab, a 450 million years old species, where A2M comprises the main constituent of the crab´s innate immune system [48].

Although characterized by significant oxidative stress, the NMR does not show age related susceptibility to oxidative damage or increased ubiquitination [12].

Recently, a high level of cysteine was found in NMR in connection with a remarkable resistance to protein unfolding [4]. In this line, it is important to note that A2M has been discussed as an important chaperon to prevent protein aggregation [49].

A number of genes associated with oxido-reduction were strongly over-represented in the NMR liver, which could convey protection against reactive oxygen species [12]. In addition, a number of enzymes and proteins involved in anti-oxidative response are zinc-dependent. Zinc is not only required for catalytic functions e.g. in superoxide dismutase but it also stabilizes protein structures such as transcription factors, hormones and hormone receptors [50]. Within cells zinc is stored in metallothioneins, but the transport in blood is only carried out by A2M [13]. A2M binds zinc with high affinity and replenishes the intracellular stores via LRP1-mediated endocytosis. Zinc is crucial for immune efficiency during aging and aging-related diseases. Thus, high levels of A2M in NMR may be important to maintain zinc homeostasis [51]. In this line it is important to know that mainly elderly people suffer from a zinc deficiency, which comes along with an immune deficiency and other age-related diseases [52–54].

Last but not least, the concept of renewal, diminishing aging-related impairments and foster longevity was recently shown by serum replacement experiments in mice. Substitution of blood from adult mice by serum from young animals lead to improved cognition, learning and memory functions probably by activation of cyclic-AMP response element binding protein (Creb) in the aged hippocampus [55]. Whether A2M is involved in such mechanisms is elusive but warrants consideration.

## Conclusion

To know the driving forces and molecular mechanisms leading to cancer resistance, longevity and diminished age-related morbidity in the NMR is of global interest. We hypothesize that A2M due to its high concentration in NMR and its vital role in a variety of fundamental biological functions seems to be a candidate to link aging, cancer and redox homeostasis in the NMR. Future research will illuminate the molecular pathway of NMR-A2M to affect cell and tissue homeostasis and its possible functions to promote cancer resistance and longevity.

## Material and Methods

### In silico sequence analysis

Comparative analysis of human and NMR-A2M mRNA and protein sequences were done by BLAST. Prediction of N-glycosylation sites in the NMR-A2M was done with the Expasy glycomod tool (http://web.expasy.org/cgi-bin/glycomod/glycomod.pl) and prediction of trypsin cleavage sites was done with the Expasy peptide cutter tool (http://web.expasy.org/peptide_cutter/). Phylogenetic tree analysis was done with ClustalW2 (https://www.ebi.ac.uk/Tool/phylogeny/clustalw2phylogeny).

### Material

Human A2M (#04–02), LRP1 (#04–03), rabbit polyclonal anti-human A2M antibody (#01–01), monoclonal anti-human A2M antibody (alpha-1; #02–02), monoclonal anti-human LRP1-beta-chain antibody (#02–04), methylamine-treated A2M (A2M-MA) (#05–04) were purchased from BioMac (Leipzig, Germany). The receptor-associated protein (RAP) was a gift from BioMac (Leipzig, Germany). A monoclonal anti-human EpCAM antibody (sc-25308) was purchased from Santa Cruz Biotechnology (Dallas, Texas, USA). A monoclonal anti-human CD29 (4706) and CD44 (3570) antibody were purchased from Cell Signaling (Leiden, The Netherlands). HRP (horseradish peroxidase)-labeled goat anti-mouse Dako, Hamburg, Germany) and–labeled anti-rabbit antibody (Jackson ImmunoResearch Lab, West Grove, USA) were used as secondary antibody. Nα-benzoyl-L-arginine p-nitroanilide (BAPNA) was from Sigma-Aldrich (Taufkirchen, Germany). Bovine serum albumin (BSA) was obtained from Serva (Heidelberg, Germany).

### Fibroblast cell culture and trypsination assay

Fibroblasts were cultured in Dulbecco's modified Eagle's medium supplemented with 10% FCS as described previously [56]. Fibroblasts were grown to 70% confluence in 24-well plates and treated with 0.3% or 1% activated (methylamine treated) NMR plasma [26]. Controls were treated with PBS or 1% human plasma for 24 h. After incubation cells were washed with PBS two times, treated with trypsin/EDTA (Life Technologies, Darmstadt, Germany) for 1 min at 37°C, washed with PBS and the remaining cells at the well’s bottom were fixed with 5% formaldehyde/PBS for 10 min, washed several times with PBS, incubated with 1% gentian solution (Sigma-Aldrich, Taufkirchen, Germany) for 10 min, washed with water, followed by extraction with 250 μL 33% acidic acid (Carl Roth, Karlsruhe, Germany) and measurement of the absorbance at 590 nm. Thereby, the colour intensity of the acidic acid extraction relates to the number of adherent cells after trypsination.

Fibroblast used for protein extraction were cultured to 70% confluence in 75 cm^2^ flasks and treated with 0.3% or 1% methylamine treated NMR plasma. Controls were treated with PBS or 1% methylamine treated human plasma for 24 h, washed with PBS followed by the protein extraction procedure.

### Blood plasma

Human heparin plasma was obtained from male healthy volunteers in the age of 30 to 40 years. All participants provide their written informed consent to participate in this study. The local ethic committee of the Faculty of Medicine of the University of Leipzig, Germany, approved this study in accordance to the ICH-GCP guidelines (reference number: 057-2010-08032010). NMR heparin plasma was obtained from 5 adult animals (2–3 years) from the Leibniz Institute for Zoo and Wildlife Research, IZW (Berlin, Germany). All animal studies were carried out in strict accordance with the recommendations for the care and use of animals and were approved by the local ethics committee of the “Landesamt für Gesundheit und Soziales”, Berlin, Germany (reference numbers: #ZH 156 and G02217/12).

### Polyacrylamide gel electrophoresis

Between 3 to 30 μg of plasma proteins were loaded either to native polyacrylamide gradient gels (native PAGE; 4%-20%) according to Birkenmeier et al. [26] or to homogeneous polyacrylamide gels (7%) containing SDS (SDS-PAGE) under reducing or non-reducing conditions followed by Coomassie Blue R 250 staining [57]. In indicated cases SDS-PAGE was run also in polyacrylamide pore gradient (4%-20%). Native, methylamine- and trypsin-transformed A2M plasma samples were separated by rate electrophoresis as previously described [26, 28]. Determination of protein concentration was performed according to Bradford [58] using bovine serum albumin for calibration.

The rate electrophoresis was done with native human or NMR plasma, and with methylamine (MA) or trypsin treated plasma. Briefly, A2M in human and NMR plasma was transformed by treatment with 0.1 M methylamine (Sigma Aldrich, Taufkirchen, Germany) for 2 h at room temperature followed by dialyses against PBS at 4°C overnight. Trypsin (Mucos Pharma, Berlin, Germany) treatment was done by incubation of plasma or A2M with twofold molar excess of proteinase over A2M for 2 min at room temperature followed by adding the proteinase inhibitor PMSF to 1 mM. For electrophoresis 30μg of protein was loaded to a RATE gel to separate the two forms of A2M, the fast-moving (transformed A2M or A2M*) and slow-moving form (native A2M).

### Liver and fibroblast cell extracts

The liver extracts from mice, human NMR were prepared by mechanical homogenization of the organs followed by incubation with extraction buffer (25mM Tris, 2 mM EDTA, 2mM DTT, 1 mM PMSF, 10% glycerol 1% Triton X-100, pH 8) containing 0.3% proteinase inhibitor cocktail (P8340, Sigma-Aldrich, Taufenkirchen, Germany) for 30 min on ice. Fibroblast cell extract was prepared by incubation of adherent washed cells (in 75 cm^2^ flasks) with 500 μL extraction buffer and additives (see above) for 15 min at 37°C and scraped by a cell scraper. After spinning for 15 min at x 13000 rpm the supernatant was then analyzed for protein content and subjected to electrophoresis.

### Immunoblotting

Plasma proteins were separated by SDS-PAGE and blotted to a nitrocellulose membrane. Unspecific binding was blocked with 5% defatted milk in TBS-T (Tris-buffered saline; 50 mM Tris, 150 mM NaCl, 0.5% Tween-20, pH 7.5) for 2 hours at room temperature (RT). The membrane was incubated with either the polyclonal rabbit anti-human A2M (1 μg/mL), the monoclonal mouse anti-human A2M antibody alpha-1 (5 μg/mL), the monoclonal mouse anti-human LRP1 antibody (10 μg/mL) and the monoclonal anti-human EpCAM antibody (0.8 μg/mL) in 0.5% milk/TBS-T overnight at 4°C. Detection was done with a HRP-labeled goat anti-mouse (1:5000) or-anti-rabbit antibody (1:7500) in 0.5% defatted milk/TBS-T for 2 hours at RT. Immunoreactive signals were visualized by enhanced chemiluminescence detection (Merck Millipore, Darmstadt, Germany). Due to the lack of appropriate antibodies and the inter-species variability to find a protein which is equally distributed in NMR und human to serve as reference gene, we decided to show a representative section of the corresponding Ponceau S staining of the blotted membrane.

### Receptor binding assay

The binding of A2M from human and NMR plasma to its receptor (LRP1) was performed by spotting 100 ng purified human LRP1 and 100 ng BSA (negative control) respectively to nitrocellulose membrane. The membrane was dried and blocked with 5% milk powder in buffer (20 mM HEPES 150 mM NaCl 5 mM CaCl_2_x2H_2_O 1 mM MgCl_2_x6H_2_O) for 2h at RT followed by incubation by 10 μg/mL of human or NMR plasma overnight at 4°C. After three washings with the above-mentioned buffer the membrane was incubated with the polyclonal rabbit anti-human A2M antibody (5 μg/mL) and the HRP-labeled goat anti-rabbit- antibody (1:7500) for 2 h at RT. Immunoreactive signals were visualized by enhanced chemiluminescence detection. The binding was also analysed in the presence of 1.5 μM inhibitory receptor-associated protein (RAP) added 30 min prior of the specific plasma samples.

### Tryptic and anti-tryptic plasma activity

The proteolytic activity of plasma corresponding to 2.5 μg to 100 μg plasma protein was measured in 50 mM Tris/HCl, pH 8.0 by following the cleavage of BAPNA to Nα-benzoyl-L-arginine and p-nitroanilide at 405 nm.

For detection of the anti-tryptic activity a plasma sample corresponding to 2.5 μg to 100 μg of NMR or human plasma protein were incubated with 0.05 μg trypsin dissolved in 1 mM HCl for 10 min at RT and the turnover of BAPNA was measured. The tryptic intrinsic activity of the plasma samples was measured and subtracted.

### Statistics

All analyses were done using GraphPad Prism 5.0 (GraphPad Software Inc. San Diego, USA). If not stated otherwise, all statistical tests were made by Mann-Whitney test. P<0.05 had been considered as significantly different.
